# Development of Wireless Sensor Network for Environment Monitoring and Its Implementation Using SSAIL Technology

**DOI:** 10.3390/s22145343

**Published:** 2022-07-18

**Authors:** Shathya Duobiene, Karolis Ratautas, Romualdas Trusovas, Paulius Ragulis, Gediminas Šlekas, Rimantas Simniškis, Gediminas Račiukaitis

**Affiliations:** 1 Department of Laser Technologies, FTMC—Center for Physical Sciences and Technology, Savanoriu Ave. 231, LT-02300 Vilnius, Lithuania; karolis.ratautas@ftmc.lt (K.R.); romualdas.trusovas@ftmc.lt (R.T.); gediminas.raciukaitis@ftmc.lt (G.R.); 2 Department of Physical Technologies, FTMC—Center for Physical Sciences and Technology, Sauletekio Al. 3, LT-02300 Vilnius, Lithuania; paulius.ragulis@ftmc.lt (P.R.); gediminas.slekas@ftmc.lt (G.Š.); rimantas.simniskis@ftmc.lt (R.S.)

**Keywords:** Internet of Things, wireless sensor network, SSAIL technology, web server

## Abstract

The Internet of Things (IoT) technology and its applications are turning real-world things into smart objects, integrating everything under a common infrastructure to manage performance through a software application and offering upgrades with integrated web servers in a timely manner. Quality of life, the green economy, and pollution management in society require comprehensive environmental monitoring systems with easy-to-use features and maintenance. This research suggests implementing a wireless sensor network with embedded sensor nodes manufactured using the Selective Surface Activation Induced by Laser technology. Such technology allows the integration of electrical circuits with free-form plastic sensor housing. In this work, a low-cost asynchronous web server for monitoring temperature and humidity sensors connected to the ESP32 Wi-Fi module has been developed. Data from sensor nodes across the facility are collected and displayed in real-time charts on a web server. Multiple web clients on the same network can access the sensor data. The energy to the sensor nodes could be powered by harvesting energy from surrounding sources of electromagnetic radiation. This automated and self-powered system monitors environmental and climatic factors, helps with timely action, and benefits sensor design by allowing antenna and rf-circuit formation on various plastics, even on the body of the device itself. It also provides greater flexibility in hardware modification and rapid large-scale deployment.

## 1. Introduction

Wireless sensor networks (WSNs) are becoming increasingly common in various areas, including industry, transportation, environment, and healthcare. Furthermore, the growing use of Internet of Things (IoT) technologies in almost all sectors [1] supports this trend. The primary focus of this research study was on the embedded system of sensor nodes using Selective Surface Activation Induced by Laser (SSAIL) technology [2,3] for temperature and humidity monitoring. Using this technology, antenna and rf-harvesting circuits can be formed on various plastics, including the device’s body. As a result, the device can be made smaller. The communication module that supports energy-harvesting antenna circuits enables the rapid, cost-effective fabrication of three-dimensional circuits on flexible material. Furthermore, even in harsh conditions, the long-term operation of distributed sensors can be achieved by collecting energy from various electromagnetic radiation sources [4]. In addition, collecting energy from the environment can reduce the need for sensor network maintenance by ensuring that they have sufficient energy for a longer lifetime, resulting in many convincing environmental and deployment benefits [5,6].

The rapid development of IoT devices has resulted in various embedded web server possibilities. Current technological advancements in industrial wireless monitoring networks (IWSNs) primarily deal with applications from emergency systems, regulatory control systems, supervisory systems, open-loop control systems, alerting systems, and monitoring systems [7]. In contrast, the installation of large-scale IoT devices complicates the use of control systems in production lines because their huge size and delicate structure make them more difficult. Therefore, one of the most affordable and widespread solutions to society’s needs has been contributed through this research. The sensor system could be customised using software and hardware technologies, making it easy to deploy for environmental monitoring and providing an option for energy harvesting to power the node in locations that are sufficiently abundant with ambient RF signals for extended periods of time.

Analysing the characteristics and parameters of sensor network nodes distributed randomly saves a lot of time and money for the real-time deployment of IoT devices on a large scale. An architecture for the master and slave sensor nodes has been proposed for the monitoring system. The communication module and energy-harvesting antennas were designed and tested. The proposed sensor node’s power consumption has been evaluated to ensure its long-term operation. An investigation into one of the commonly used communication protocols was carried out, and the findings were reported. The total power consumption of each sensor node was measured using a deterministic operating regime. The monitoring of the sensor nodes was accomplished independently through the high-level hosting application. The utilisation of solar and wind energy as a source of energy harvesting would be the superior choice in more remote open locations. WSNs built by this approach can be used to monitor the forest, including unmapped areas after installation.

## 2. Related Work

This section provides an overview of the most recent advancements that have been made in the application of WSNs. Precision agriculture, industrial, forestry, and weather monitoring systems can all benefit from the suggested method. Industry 4.0 categorises numerous wireless sensor networks [7,8] coverage approaches, deployment issues, sensing models, and research obstacles, including sensor networks, fog, edge computing, distributed control systems, digital twins, and cyber–physical systems. Due to the size and adaptability of our system, it is able to support the rapid deployment of IoT devices on conveyor belts for the purpose of monitoring the process of production lines or various areas for environmental monitoring.

The IoT application for forestry management involves data transfer to cover locations where 3G/4G signals are unavailable. The authors used low power wide area network (LPWAN), NB-IoT (NB—narrow band), LoRa (long-range), and appropriate sensors to convey data [9]. Using that approach, they employ two repeaters to cover the more considerable distance. In addition, the sensor network that they have works well for measuring air pressure, UV light, and carbon dioxide on hillsides. In light of the findings from earlier research, the decision was made to install retransmitters to cover the distance inaccessible to communication channels.

Atmospheric environmental monitoring plays a major role in monitoring the aerosol, particulate matter, haze, dust storms, straw burning, and gaseous pollutants that contribute to global warming. Three types of atmospheric monitoring use remote satellite sensing, and improved retrieval methods, product validation, local application, air quality monitoring, and control are all part of the network [10]. However, according to experts, the current monitoring precision is insufficient to meet the management demands of the atmospheric environment.

A portable weather-monitoring system for greenhouses to measure temperature, humidity, and moisture is presented in [11]. The solution provides a simple gadget that could measure such characteristics reliably and practically. The gathered sensor values are regularly updated using the ThingSpeak IoT’s web service. However, this approach is unreliable in a larger region. The proposed system in our work using the PHP application and MySQL database results in easy time-series management and can forecast future sensor values in the web server, and the real-time charts can display a maximum of 150 data points.

Traditional farmers gain a valuable technological tool by using the IoT to monitor and control environmental variables in a coffee crop via a wireless sensor network. This allows them to increase their economic benefits, reduce environmental impact, and improve the quality of life. The Zigbee protocol by Zigbee Alliance was suggested in [12] over other wireless technologies with equivalent capabilities because of its low cost. However, due to the excessive humidity in coffee plantations, the failure to execute all of the anticipated measurements causes difficulties with the sensors and the readings of this monitoring system.

The energy optimisation of the quality of service (QoS) in wireless sensor networks is highly essential and depends on different routing protocols such as the RPAR (real-time power-aware routing) protocol, GRPAR (greedy real-time power-aware routing) protocol [13], and the low-energy adaptive clustering hierarchy (LEACH) protocol. The latter is based on clustering, and the nodes can only send to the cluster head (CH). Since WSNs interact with the environment, their characteristics are expected to differ from conventional data networks. QoS support for the network has to consider a few of the unique challenges such as severe resource constraints, unbalanced traffic, data redundancy, network dynamics, energy balance and scalability, multiple sinks, traffic types, and packet criticality. When the application of the sensor network is specified, QoS helps support and overcome the challenges mentioned above.

Firstly, the applications that run on WSNs are not end-to-end applications like they used to be. Secondly, the bandwidth is not the main concern for a single-sensor node [14]. However, the bandwidth may be an essential concern for a group of sensors for specific periods due to the burst nature of sensor traffic. Thirdly, packet losses in traffic generated by one sensor node can be tolerated to a certain extent, since there is always redundancy in the data. Finally, most applications in WSNs are mission-critical, reflecting the importance of applications. Consequently, they are confident that the QoS support in WSNs cannot be gauged using end-to-end network QoS metrics alone. Thus, there is a knowledge gap for non-end-to-end QoS parameters that must be proposed. QoS parameters are collectively called “collective QoS parameters”, referring to these non-end-to-end characteristics. These are collective latency, collective packet loss, collective bandwidth, and information throughput [14].

The hybrid cluster approach for homogeneous and heterogeneous networks to increase the QoS parameters of wireless sensor networks based on MATLAB simulation results is devised in [15]. Based on throughput and network lifetime parameters, the proposed WSN QoS-based energy-efficient protocol outperforms ATEER (average threshold energy-efficient routing), DEEC (developed distributive energy-efficient clustering), and EDDEEC (enhanced developed distributive energy-efficient clustering) by 10%, 26%, and 63%, respectively. The study concludes that the protocol based on throughput and network lifetime parameters is well suited and well positioned for designing WSNs in real-world and real-time scenarios.

In the node status and score-based route optimisation protocol (NSSROP), each node stores some additional data to balance the routing burden among all nodes [16]. A sensor node may have the shortest route to a base station in an IoT configuration. The WSN’s block-listing method is established to cope with non-cooperative nodes and the NSSROP has achieved exceptional results in terms of average energy usage, throughput, and end-to-end delay [16]. Unlike other standard routing protocols, the proposed technique considers various QoS criteria related to routing and energy optimisation in WSNs. To determine the node densities, each node is evaluated based on its energy consumption and closed neighbours (CNs).

A hybrid method for obtaining the probability distribution of report latency in random access (RA) WSN protocols [17] is studied and it is beneficial for assessing the QoS of WSNs in time-critical applications. In this manner, event reporting is time-constrained and fault-sensitive. In specific applications such as target tracking and positioning, transmitting a certain number of event packets is required to characterise the occurring phenomena accurately. Our methodology considers a basic structure, and the distribution of the number of detecting nodes is obtained by simulation, allowing the analysis of the desired QoS parameters.

Most WSN technologies are designed to monitor and manage various processes effectively. A “smart environment” is equipped with sensors, microcontrollers, and adaptive software programs for self-protection and self-monitoring. For designing a WSN, several factors need to be considered. Such factors include scalability, fault tolerance, high sensing accuracy, inexpensive deployment costs, rapid scaling up, and application needs. The sensor networks will become an integral part of our lives in the future because of their wide range of applications. However, before sensor networks can be implemented, constraints such as cost, hardware, topology alteration, environment, and power consumption must be addressed. In addition, sensitive networks demand particular wireless ad hoc networking protocols because of the tight restrictions.

Our methodology of designing the wireless sensor network monitoring for the environment using SSAIL technology focused on inexpensive deployment cost, rapid scaling up, and application in the production lines of factories. Since this system allows for the installation of any type of sensor, it can also be used for outdoor monitoring. Our process of designing a sensor node contributes:

✓ The hardware modification of the communication module, providing the opportunity to adopt the RF-harvesting antenna circuit;
✓ The design of a fractal antenna for RF-harvesting applications, and the investigation of simulation vs. measurement antenna characteristics;
✓ The simulation of randomly distributed sensor nodes to find the position of the sensors according to the parameters of area length, width, expected sensor reading per minute, and size of the data packet. The detached nodes and the communication path are analysed;
✓ The sensor node architecture and its specific operational regime;
✓ The power consumption of the communication module and its mode of operation;
✓ A simulation of the improved LEACH protocol compared with the standard LEACH protocol, which is analysed for its lifetime of the sensor node, total energy dissipated, and the throughput;
✓ The estimation of the overall energy consumption per day of the analysed network consisting of 10 sensor nodes;
✓ The hosting of high-level PHP applications using the MySQL database and data visualisation in real-time charts on the web server.

## 3. Materials and Methods

Our research was concentrated on creating the different layers of technology that make up the sensor network protocol stack, energy harvesting, efficient routing algorithm, and an asynchronous web server for environmental monitoring to meet the demand for a real-time IoT monitoring solution.

### 3.1. Methodology

The communication module was designed using SSAIL technology. On a physical level, the 3D MID concept (electronics embedded in free-shape polymeric components) could aid the sensor network deployment [18]. This technology utilising laser treatment and selective electroless metal deposition is a promising approach to producing sensor nodes and energy-harvesting systems combined in a single body, allowing for more shape, size, and material freedom [2,3]. The IoT network for temperature and humidity monitoring using an asynchronous web server are customized as follows:

- Design and deployment of an asynchronous web server using the following technologies: ESP32 programmed with Arduino IDE, hosting server and domain name, and hypertext pre-processor (PHP) script to insert data into the MySQL database and display it on the web page in charts.
- Estimation of the power consumption of a sensor node and analysis of its performance during various operating modes.
- Prototyping the sensor node with data management and energy-harvesting subsystems.

**Figure 1:**
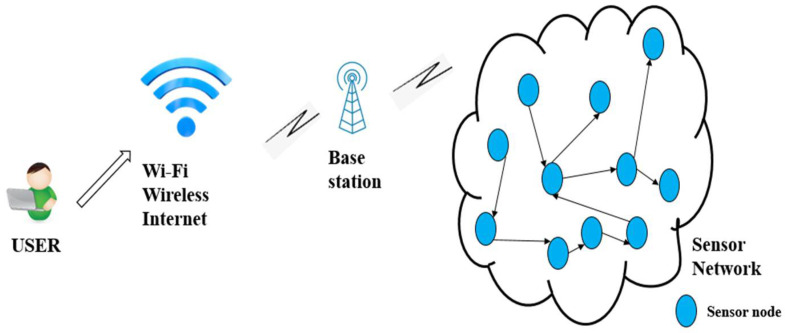
Scheme of typical WSN with multiple nodes.

A user communicates through the internet with a base station directly or indirectly connected with all sensor nodes distributed in the area to be monitored. Figure 1 depicts a typical design of a WSN [19].

### 3.2. Wi-Fi Module and Sensor Node Design

The microcontroller ESP32 from Espressif systems is a low-cost and low-power microcontroller with dual-mode Bluetooth and Wi-Fi that uses Tensilica Xtensa LX6 microprocessor dual-core and single-core varieties. The module can function as both a client and a server, and operates at a low 3.3 V power supply.

**Figure 2:**
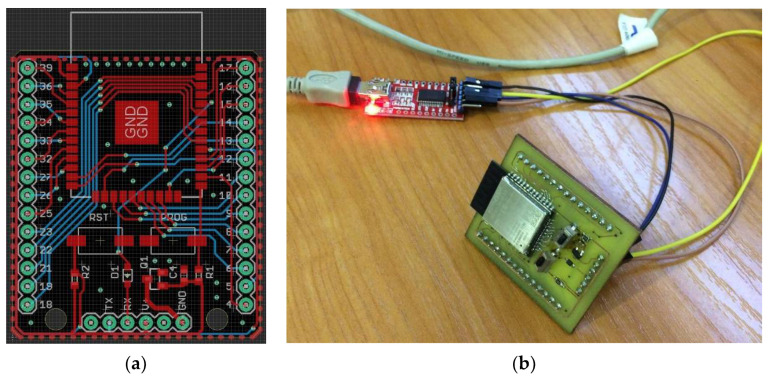
Design of PCB board (**a**) and modified ESP32 module prototype manufactured using SSAIL technology (**b**).

The flexible nature of the SSAIL technology allows it to produce electric conductor lines on any dielectric surface, allowing it to adapt to the geometry of the network node. The circuit for a Wi-Fi module was designed using the Eagle software. AutoCAD was used to make minor adjustments to the circuit design layout to guarantee that the laser machine would produce the appropriate hatch during subsequent processing. The procedure involves using an ultrashort-pulse laser to modify the surface of the FR4 glass-reinforced epoxy laminate, chemical activation of laser-modified regions, and electroless metal deposition on the locally activated surface. Figure 2a depicts the PCB layout of the ESP32 module that performs all of the functions of the sensor node.

Figure 2b shows the setup of an assembled PCB with a modified module prototype manufactured using SSAIL technology connected to a flawless FTDI FT232 series USB interface IC, used to upload programming code to the Wi-Fi module using Arduino IDE. The modified communication module replaces the standard module, which cannot support the energy-harvesting antenna in WSNs. In addition, Adafruit DHT11 sensors were connected to an Espressif ESP-Wroom32 microcontroller, which runs an asynchronous web server for temperature and humidity monitoring. For preliminary testing, the modified prototype batteries were used. When compared with the performance of the standard ESP32 module, the results of the modified prototype’s performance are extremely encouraging towards our goals of enclosing the antennas together with it.

Energy-harvesting antennas can be used instead of a power unit in sensor nodes that are compact enough to carry the needs of the sensor nodes. To meet the Wi-Fi module’s power consumption, the designed antenna must be compact and sufficient. A compromise approach to integrating the communication module with a modified power unit using SSAIL technology has been proposed by the researchers. The sensor node’s compact structure allows it to be installed in a small area, such as a production conveyor, for monitoring purposes, which is absolutely necessary as research develops.

### 3.3. Sensor

In the initial phases of research, the Adafruit Industries LLC DHT11 digital temperature and humidity monitoring sensor, which detects critical environmental characteristics such as temperature and humidity, is utilised. It uses a capacitive humidity sensor and a thermistor to monitor the ambient air before releasing digital data to the output (no analogue information pins are required). The sensor runs at 3–5 V and can detect air humidity between 20% and 80% with 5% precision and temperature between 0 °C and 50 °C with a precision of 2 °C.

**Figure 3:**
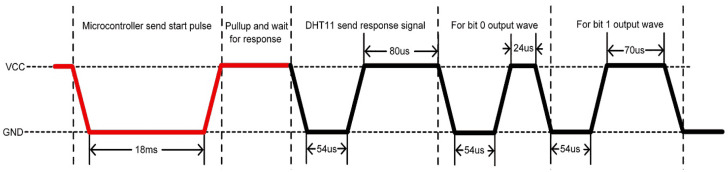
Communication cycle of the sensor.

The communication process with the microcontroller is divided into three steps. The first is to send a request to the DHT11 sensor. The sensor sends a response pulse back, and then it sends data totalling 40 bits to the microcontroller, as shown in Figure 3. After delivering 40 bits of data, the DHT11 sensor has a 54 µs low level and then goes high. It goes into sleep mode after this.

### 3.4. Design of Energy-Harvesting Antenna

Electronic circuits may be directly integrated on 3D shaped dielectric substrates, making moulded interconnect devices (MID) a feasible solution for IoT applications [18]. The sensor nodes were realised using the SSAIL technique [2,3]. Interconnections with excellent conductivity and resistance to extreme environmental conditions were created using electroless copper, nickel, and gold plating on the laser-activated surface. The sensor node electronics were integrated inside the moulded block.

**Figure 4:**
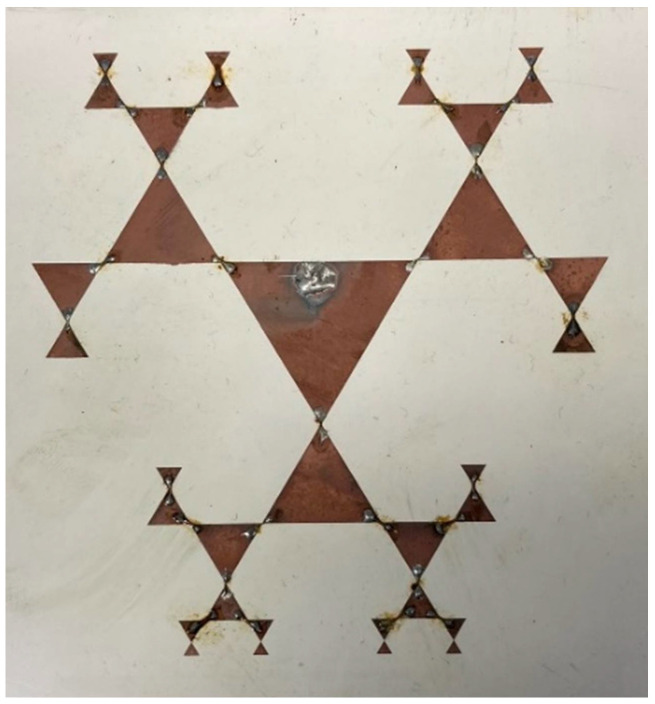
Energy-harvesting antenna.

Multiple designs of patch and fractal antennas for energy harvesting, their matching circuits with a rectifier, and rectifier schemes have been schematically modelled and experimentally investigated. The antennas were formed on various polymer substrates with different dielectric constants using SSAIL technology to form electrical conductivity tracks. The antenna shape is based on fractals taken from the website [20]. It is similar to the well-known Sierpiński gasket. It is shown that such a type of antenna made on a 1.6 mm FR4 substrate has five different resonant frequencies [21]. Figure 4 shows the designed and manufactured fractal antenna.

However, the dimensions of the antenna are too large (200 mm), and neither resonant frequency matches the Wi-Fi router’s lower working band. To adapt to the characteristics of the antenna Wi-Fi band, the antenna geometry on the PREPERM^TM^ PPE440 plastic, which has a dielectric constant of 4.4 and thickness of 3.2 mm, was optimised. For optimisation, the FDTD solver, which simulated the main characteristics of the antenna, such as S_11_ and gain, was used. The following were determined to be the optimal dimensions for the fractal antenna: the edges of the larger triangle should be 30 mm, while the edges of the smaller triangle should be 18 mm. The ratio between the edges of the smaller and larger triangles is 0.6. Therefore, the size of the whole antenna on the PPE440 substrate is 120 mm × 120 mm. The excitation port is shifted down from the tip of the larger triangle by 23.5 mm and sideward by 1.5 mm. The other side of the substrate is grounded with a continuous copper layer. Theoretical calculations have shown that such an antenna has resonances at 2.43 GHz and 5.78 GHz, where S_11_ is −30.3 dB and −28.3 dB, respectively, and the maximum gain reaches 6.3 dBi and 4.33 dBi.

### 3.5. Sensor Node Architecture and Implementation

**Figure 5:**
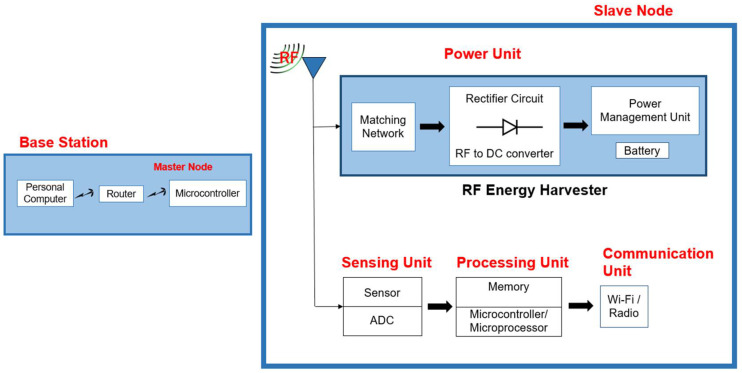
Sensor node architecture.

The master node is a so-called base station that includes a computer, router, and microcontroller. Other slave sensor nodes are linked to the master node. As illustrated in Figure 5, each slave sensor node comprises a sensor, microcontroller, and power unit. The sensor collects data from the environment and provides it to the microcontroller for processing. The slave node sends the data read from a sensor to the master node for additional processing and storage for real-time monitoring. Finally, the data received by the master node are saved on a personal computer for further study and timely action. The power unit could compromise the energy harvester to collect electromagnetic radiation for charging the battery. Experiments were conducted by employing four sensor nodes, one of which served as a master node and others are slave nodes.

### 3.6. Sensor Node Monitoring

The sensor node shown in Figure 2b was programmed using the Arduino IDE, enabling programming and uploading files to the ESP32 Wi-Fi module. Web browsers on smart gadgets, also referred to as web clients, are the responsibility of an asynchronous web server. The communication between the web client and the web server, the hosting server and the domain name is established by the Hypertext Transfer Protocol (HTTP). Web clients can connect to the web server through a router and the internet. The required activities are carried out using a standard client–server architecture and building a client that requests a PHP script to publish sensor readings in a MySQL database. The webpage displays sensor readings in a plot that can be accessed anywhere. With the assistance of this method, sensor data can be transmitted from websites to web clients. These data may then be utilised to evaluate, investigate, and take necessary action about ambient temperature and humidity variables. The following technologies to construct an optimised communication model are utilised:

- Arduino IDE, to programme the ESP32;
- Domain name and hosting server;
- PHP script for inserting data into the MySQL database and displaying it on a web page;
- MySQL database for storing readings;
- PHP script for plotting data from the database into charts.

**Figure 6:**
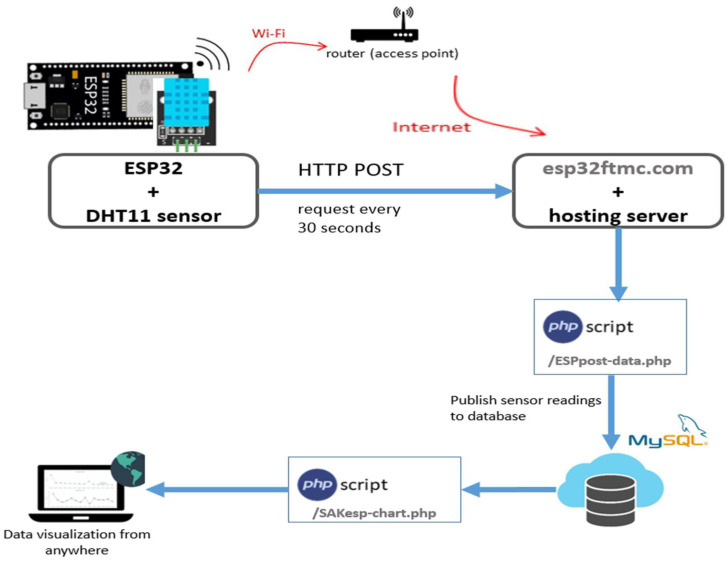
Overview of hosting PHP application and MySQL database.

The program’s design includes a HyperText Markup Language (HTML) structure that specifies how the webpage should be displayed. The ESP32 must connect to the appropriate sensors to monitor the environment to complement successfully uploading the programming code. This idea aims to have a domain name and a hosting account that allows us to store sensor readings from the ESP32. It can visualise the readings from anywhere by accessing its own server domain. To build the charts, the Highcharts library was used. It shows two charts: temperature and humidity over time. The charts display a maximum of 70 data points, and a new reading is added every 30 s. The capability of data points can be changed at any time in the code. The web client may access the data through a web browser thanks to the design of an Internet of Things network for temperature and humidity monitoring and its implementation using a web server. Figure 6 depicts the high-level overview of hosting PHP applications and MySQL database.

The proposed IoT-based temperature and humidity monitoring framework comprises sensor nodes with an ESP32 Wi-Fi module and a DHT11 temperature and humidity sensor with a power supply. In this research, the ESP32 microcontroller with a dual-core processor, and Wi-Fi connectivity was used. The sensor nodes connect with a base station either directly or via Wi-Fi-enabled nodes near the base station. The base station provides connectivity to the internet. Sensor data can be obtained via connecting to the internet. Client–server architecture is used to perform the essential task.

## 4. Results

This section summarises the simulation result of analysing the characteristics and parameters of sensor network nodes distributed randomly, power consumption analysis, and other factors compared with simulation using improved LEACH protocol and standard LEACH protocol, the analysis of the power consumption of the communication module, the results and discussions of the energy-harvesting antenna, the overall power consumption of the sensor node, and the web server monitoring system results.

### 4.1. Sensor Network Node Simulation

To analyse the real-time deployment of a sensor network in different aspects of increasing the number of nodes, area length, and width, a simulation model of sensor network nodes was constructed using MATLAB. The sensor readings from time to time, as well as the size of the data packet, can be predicted using the simulation of two alternative numbers of nodes, 20 and 80, in the area to simulate the wireless sensor network. The detached sensor nodes communicate with other nodes or the base station within their working distance. Therefore, the nodes located between the base station and other nodes could be used as retranslators.

**Figure 7:**
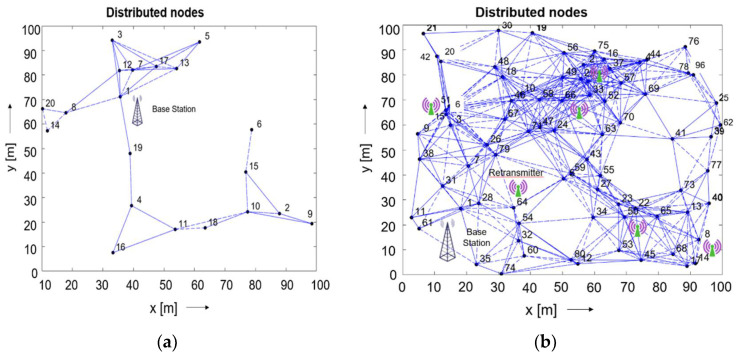
Distributed number of nodes, 20 and 80, in (**a**,**b**), respectively. Area length = 100 m, area width = 100 m, expected sensor reading = 100, and size of the data packet = five bytes.

Figure 7 illustrates a sensor network with randomly distributed nodes. Additional criteria considered for the networking simulation include area length and width, projected sensor readings per minute, and data packet size. The solid blue lines represent the forward communication, and the dotted lines represent the acknowledgement sent back. The best location the base station and retransmitting nodes are shown.

As seen in Figure 7a, the information communication path to the base station is relatively easy when a smaller number of nodes is used. When the distance to the base station is short, the network does not use any of the intermediate nodes. However, some nodes are out of reach of the base station, which only communicates with it through the intermediate nodes. Therefore, nodes with longer working distances should be applied for a reliable connection. Figure 7b shows that when the number of nodes is high, there are many ways to transfer the information to the base station. In this particular case, retranslators are planned to implement in multiple places to reduce the workload of the nodes.

When the nodes have multiple tasks, they are forced to be in an ON state to follow up on the communication protocols. In this case, more energy is consumed. Retranslators are a suitable solution to reduce the power consumption. They reduce the workload on the individual nodes while retaining the specific memory of previously sent data packets, increasing the lifetime of the sensor nodes. Various topologies and data acquisition for rapid deployment will be considered in future work.

### 4.2. Routing Protocol-Based Simulation and Analysis

The modified LEACH protocol used in WSNs to expand the network lifetime was considered for the power consumption analysis. In this protocol, the sensors arrange themselves in a cluster, and a single node performs as a cluster head. The cluster head finds the lowest degree of distance to the base station to decrease the power consumption in the cluster head nodes and the whole network. An improved LEACH protocol (I-LEACH) was implemented in communication and compared with the standard LEACH protocol. When the network parameters have been set, the simulation is run until all of the nodes drain their batteries for these two algorithms. Then, the network behaviour analysis is plotted, showing its lifetime and the amount of data sent. The proposed I-LEACH ensures that the elected cluster heads will be uniformly distributed over the network. Hence, there is no possibility that all cluster heads will be concentrated in one part of the network.

**Figure 8:**
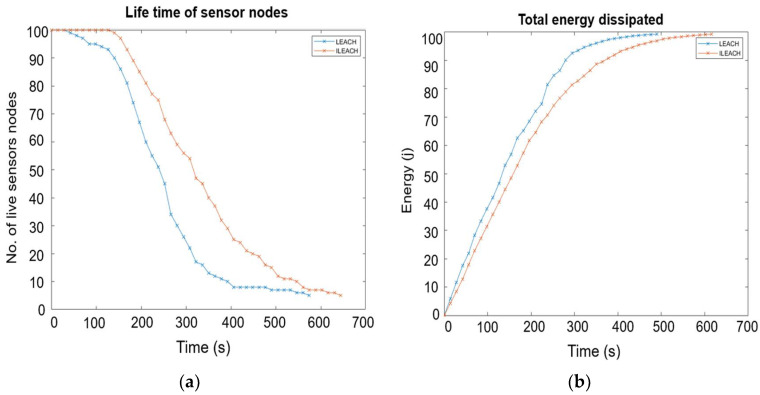
Comparisons of lifetime and average energy consumption of sensor nodes between standard LEACH and improved LEACH protocol in (**a**,**b**), respectively.

**Figure 9:**
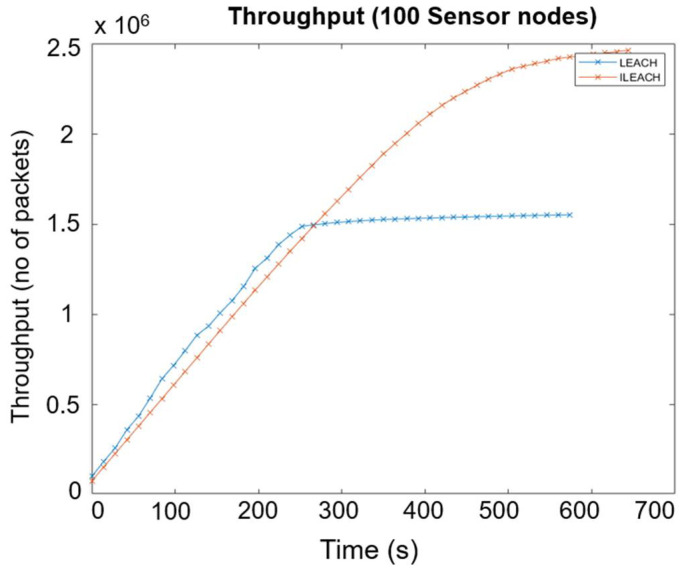
Throughput comparison between standard LEACH and improved LEACH protocol.

The simulation results indicate that the proposed clustering approach is more energy-efficient, scalable, and effective in prolonging the network lifetime compared to the LEACH protocol. Figure 8a shows the simulation result of the lifetime of 100 sensor nodes (number of live sensor nodes vs. time), Figure 8b shows the simulation result of average energy consumption (energy vs. time), and Figure 9 shows the simulation result of the throughput between the LEACH and I-LEACH of 100 sensor nodes (number of packets vs. time). At the end of the simulation, the performance of the proposed I-LEACH protocol was evaluated. The data in Figure 8b and Figure 9 prove that I-LEACH improves the energy consumption by around 43% and throughput by 40% in a 100-node network size.

Application sensor nodes will periodically switch on their sensors and transmitters, sense the environment, and transmit data of interest at a constant periodic time interval. The I-LEACH protocol is highly recommended for networking sensor nodes in real-time deployments to increase the network’s life expectancy and maintain throughput.

### 4.3. Analysis of Power Consumption by Communication Module

The power consumption was evaluated by measuring the current and voltage with a multi-meter. The ESP32 can be a relatively power-hungry device, depending on its state; the ESP32 was powered by a 3200 mAh battery in the earlier stage of the experiment. The solution here was to reduce the ESP32’s power usage by leveraging one of its sleep modes. The battery life could be dramatically extended as the node does not need to always be active. The advanced power management of ESP32 offers five configurable power modes. The chip can switch between different power modes according to the power requirement: active mode, modem sleep mode, light sleep mode, deep sleep mode, and hibernation mode. Each mode has its distinct features and power-saving capabilities.

Active mode is the normal state of ESP32. In this mode, all of the features of the chip, especially the Wi-Fi module, the processing cores, and the Bluetooth module, are ON at all times. The chip requires more than 240 mA in current to operate in this state. From the ESP32 datasheet, it is clear that the power consumption during the active power mode, with RF working, is Wi-Fi Tx packet at 13 dBm, a transmitter power of 160 mA, and Wi-Fi Rx and listening of 120 mA. This is the least efficient option and will consume the most power. To conserve power, there is a need to disable them by leveraging one of the other power modes when not in use.

Table 1 shows the various power modes of the ESP32 and their respective power consumption. Table 1 reveals that the ESP32 in the deep sleep mode could be leveraged while the device is not in use. The CPU, most of the RAM, and all digital peripherals are powered off in this mode. If the ULP coprocessor is powered on to 10 µA, the chip consumes roughly 0.15 mA.

**Figure 10:**
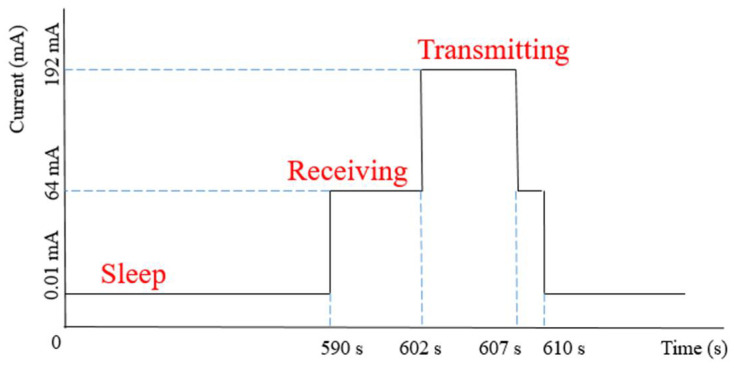
Timing diagram of sensor node operation.

For data transmission, to monitor the receiving (Rx) and transmitting (Tx) mode current, an approach of 10 min intervals was presented, and the voltage difference between the Rx and Tx mode was measured with an oscilloscope. The voltage difference (V) of 640 mV and 1920 mV for Rx and Tx modes was measured. Assuming a resistor (R) of 10 Ω (ohms) connected in series, the current consumption of 64 mA for Rx mode and 192 mA for Tx mode was calculated. Figure 10 depicts the timing diagram of the sensor node operation regime.

The sensor node is set up to run in three stages: sleep for 590 s, wake up to collect sensor data from sensors, transfer the data packet to the master node, and sleep again after the data have been delivered. Five bytes of temperature and humidity sensor data are transferred between master, intermediate, and slave nodes.

### 4.4. Harvesting Antenna

**Figure 11:**
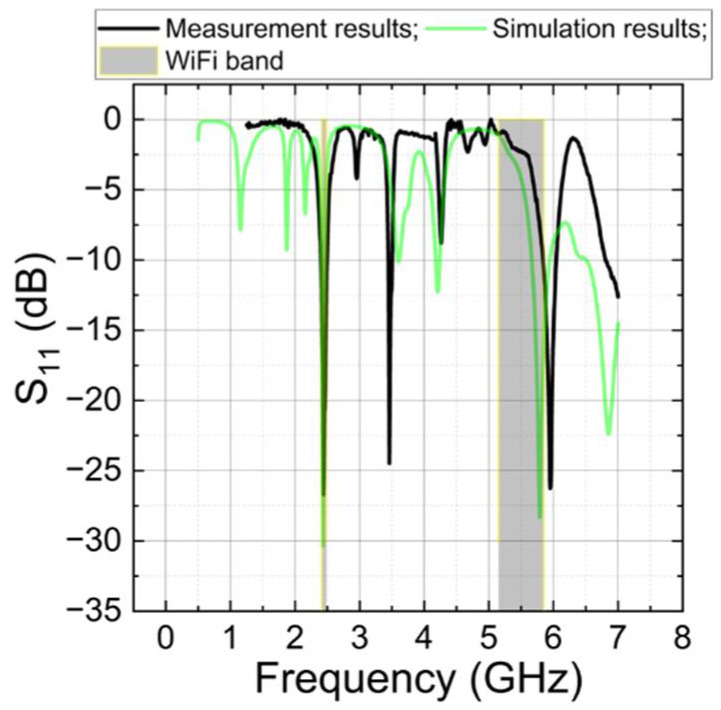
Simulation and measurement results of S_11_ manufactured fractal antenna.

For energy harvesting from the Wi-Fi network at 2.4 GHz and 5.0 GHz, a fractal antenna design was investigated. Figure 11 shows the simulation and measurement results of S_11_ of the design fractal antenna.

A comparison of the simulated and measured spectra of the antenna’s S_11_ characteristic is shown in Figure 11. The black line corresponds to the measurement results, the green line represents the simulation results, and the grey area indicates the Wi-Fi frequency bands at 2.4 GHz and 5 GHz. From Figure 11, it can be seen that there is a perfect match between the simulation and measurement results at 2.43 GHz. The measured bandwidth (at the −10 dB level) is 2.40 GHz to 2.49 GHz. This bandwidth fully covers the lower Wi-Fi frequency band. Not such a good agreement is found at higher frequencies. The expected theoretical reflection minimum at 5.78 GHz is shifted to 5.95 GHz, and the measured bandwidth for the upper Wi-Fi frequency band was from 5.8 GHz to 6.04 GHz. From Figure 11, it can be seen that only a small part of the upper Wi-Fi frequency band can be used. Nevertheless, at least nine Wi-Fi channels should fit into this frequency band. To match the spectral characteristics of the antennas to the frequencies used by Wi-Fi, the dielectric properties of the polymeric materials used in the substrates must be taken into account.

**Figure 12:**
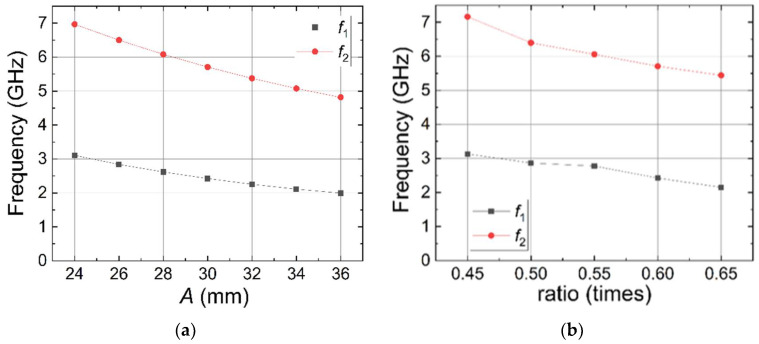
Dependence of antenna resonance frequencies on the size of the edge of the largest triangle (**a**) and dependence of antenna resonance frequencies on the ratio between edges of the fractal triangles (**b**).

Additional investigation was performed into how the working frequencies of the fractal antenna depend on its two main parameters—the size of the largest triangle and the edge ratio of other triangles. The upper limit of the parameter space is limited by the size of the PPE440 substrate. All other parameters are fixed. The simulation results are presented in Figure 12—the increase in the size of the triangles or the ratio between the triangle edges decreases the antenna resonance frequency in both bands. The dependences are close to linear. The ratio between the two frequency bands increases from 2.24 to 2.41 when *A* increases, and from 2.29 to 2.53 when the ratio between the triangle edges increases. This allows us more precise control of the resonance frequencies of both bands by choosing proper dimensions of the antenna and slight separate tuning of both resonance frequencies.

For microwave rectifying, Schottky diodes SMS7630 from Skyworks were applied. At an optimal load resistance, a voltage of about 0.5 V could be expected at the output of the rectifier circuit. The voltage is too low to power microcontrollers (3–5 V needed), and an additional power control module should be connected to the power supply to charge the battery. The power management module BQ25570, manufactured by Texas Instruments, was selected as it has a low input voltage and it has a high-efficiency factor. It can be expected that when the energy-harvesting circuit is irradiated by a maximum allowable microwave power density of 10 W/m^2^, the output of the generated energy harvester could provide a balanced power of 5 mW. The design of energy harvesters for other spectral ranges is ongoing. However, the initial results for energy collection from electromagnetic waves existing in the environment are promising. Other sorts of energy (solar, wind, etc.) could be harnessed to power detached sensor nodes in WSN. The SSAIL technology can be used to integrate any of these kinds of energy harvesting antennas with a Wi-Fi module using the appropriate design matching the requirements.

### 4.5. Estimation of Power Consumption by Sensor Node

The estimated total power consumption of the sensor network with ten nodes is presented in Table 2 below. In light of the numerous communication channels, its duty cycles have been brought up for discussion. This particular operating regime has a duty cycle of 10 min intervals.

Node 1 is closest to the base station, and node 10 is farthest from it in a star topology when there are ten sensor nodes. Nodes that retransmit are known as retranslators. There are two types of nodes in a cluster: “detached nodes” and “gateway nodes” (node 1). Node 1 connects with other nodes for acknowledgement as a cluster head and acts as a gateway node for sensor data transfer to the base station. On the other hand, detached nodes are exclusively responsible for collecting and transmitting data to the cluster head.

Based on column 4, the sensor network of ten nodes consumes 65.31 mW/day in total if the nodes send one piece of information in an hour for the selected operation regime. When this fact is considered, the lifespan of various types of batteries can be estimated. The RF energy harvester, which can serve as a primary charging battery source for a wireless sensor network, could be used to operate under multiple operating regimes.

### 4.6. Sensor Node Implementation Result

**Figure 13:**
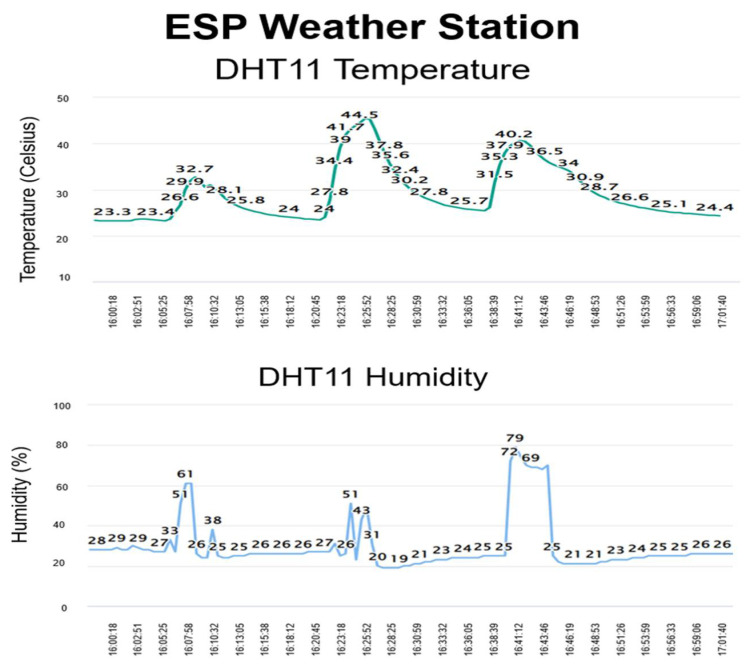
Sensor readings in real-time charts in web server accessed by web client.

The sensor readings are displayed on the server, which can be accessed anywhere. The created database shows the result of the DHT11 sensors in two real-time charts: one shows the temperature readings, and the other shows the humidity readings. Figure 13 displays the outcome of a web client monitoring the temperature and humidity in a web server.

With this setup, the user has complete control over the server and can easily switch hosts, if necessary. There are numerous free and paid cloud options available for publishing the readings, but each has its drawbacks, such as limitations on the number of readings posted, the number of linked devices, and who may see the data. Furthermore, the cloud service can be changed or discounted at any moment. The output is as simple as possible to understand, and it can be reprogrammed to modify the web page’s layout, post different sensor values, publish from numerous ESP boards, and much more.

## 5. Discussion

For the battery-free multi-node sensor system [1,4], the system was designed using SSAIL technology [2,3] and validated for harsh environment implementation scenarios. For the energy-harvesting technique [4], research challenges on topologies and the energy management system [6,13], and the proposed system optimization, would increase the system reliability and scalability. Our discussion stresses the classification of coverage [5,6,7,8], practical challenges in the deployment of WSNs [8,9,10], QoS support [15,16,17], sensing models, and research issues in WSNs [11,12].

The sensor node architecture suggested for the system consists of a base station in communication with multiple slave nodes. The slave node should be equipped with a sensing unit that contains sensors, a modified processing unit that supports the idea of enabling RF energy-harvesting circuits, Wi-Fi for communication with the master node, and the power source. In addition, a simulation model was used to investigate and compare the improved LEACH protocol’s lifetime of sensor nodes, total energy dissipated, and the throughput of 100 sensor nodes with the standard LEACH protocol. The sensor transmits five bytes of data to the Wi-Fi module operating in deep sleep mode each time it is activated. This is the first demonstration of a multi-node sensor system designed using SSAIL technology. In contrast to solutions relying on various energy sources [1,4,7,8,9,11], the power unit RF harvesting itself is integrated and designed as a component of the system, ensuring full control over the amount of available energy and the continuous operation of the wireless sensor nodes.

As a result of S_11_ fractal antenna [20] simulation and measurement, we succeeded in finding a design with the measured bandwidth that entirely covers the lower Wi-Fi frequency band. The antenna resonates at five distinct frequencies, resulting in more energy gathering features. Unlike [21], additional investigation into the working frequencies of the fractal antenna depending on the size of the largest triangle and the edge ratio of other triangles was performed. At the −10 dB level, the measured bandwidth is 2.40 GHz to 2.49 GHz. From Figure 12, clearly, the increase in the size of the triangles or the ratio between the triangle edges decreases the antenna resonance frequency in both bands. The dependences are close to linear. The ratio between the two frequency bands increases from 2.24 to 2.41 when *A* increases, and from 2.29 to 2.53 when the ratio between the triangle edges increases. This allows us more precise control of the resonance frequencies of both bands by choosing the proper dimensions of the antenna and the separate tuning of both resonance frequencies.

Our estimations show that if a WSN of ten nodes sends one piece of information in an hour, it consumes 65.31 mW/day in total for the selected operation regime. However, the energy consumption of every node during different duty cycles in conditions such as traffic types, packet loss during data transfer, and accuracy of the data are major concerns for future work.

## 6. Conclusions

This primary research focused on the embedded system of sensor nodes for monitoring temperature and humidity using electronic circuits and antennas manufactured with SSAIL technology. This technology benefits sensor design by enabling antenna and RF-harvesting circuit formation on various plastics, even on the device’s body. Thus, the devices can be miniaturised. The energy harvester and communication part of a node was designed and tested. The communication module used to support the energy-harvesting antenna circuits was designed to enable the rapid and cost-effective fabrication of circuits on flexible material in three dimensions. The simulation and measured results of the fractal antenna show a perfect match at 2.43 GHz, and it fully covers the lower Wi-Fi frequency band.

The sensor network with randomly distributed nodes was shown using a MATLAB simulation based on parameters such as area length, area width, expected sensor reading, and data packet size. Different modes of operation of the Wi-Fi module were analysed and it was concluded that the deep sleep mode uses the least amount of energy. The simulation results of the improved LEACH protocol and the standard LEACH protocol show that the I-LEACH reduces energy consumption by approximately 43% and increases throughput by 40% in a network of 100 nodes. The total power consumption of the sensor network of ten nodes was estimated to be 65.31 mW per day using the specified operation regime. Monitoring of the sensor nodes was accomplished independently through the high-level hosting PHP application and the MySQL database. The sensor data retrieved by the web client was displayed in real-time charts on the web server. Therefore, this model is promising for environmental monitoring air parameters in cities and industrial zones.

Future research will involve communication between multiple nodes from a network of sensors in diverse locations, parameter evaluations, accounting for radio channels, and real-time data collecting via web clients. In addition, with the advancements in wireless networking and sensor technology, there is an attractive potential for data aggregation, simulation, and testing of lifetime-aware routing and geographical coverage.

## Figures and Tables

**Table 1:** Power consumption of ESP32 module.

Modes	Energy Consumption
Active	528~858 mW
Modem Sleep	9.9~66 mW
Light Sleep	2.64 mW
Deep Sleep	33 µW
Hibernation	8.25 µW

**Table 2:** Power consumption of sensor network.

Nodes	If One Duty Cycle is 10 min, mW	If One Duty Cycle is 1 h, mW	Overall Consumption per Day, mW
1 (Cluster head)	8.450	1.408	33.792
2	0.845	0.142	3.408
3	0.845	0.142	3.408
4	0.845	0.142	3.408
5	0.845	0.142	3.408
6	0.845	0.142	3.408
7	0.845	0.142	3.408
8	0.845	0.142	3.408
9	0.845	0.142	3.408
10	0.845	0.142	3.408
Time synchronisation	-	-	0.845
	Overall power consumption		65.31

## Data Availability

Not applicable.
